# Genotyping cell-free tumor DNA in the blood to detect residual disease and drug resistance

**DOI:** 10.1186/s13059-014-0449-4

**Published:** 2014-08-30

**Authors:** Giulia Siravegna, Alberto Bardelli

**Affiliations:** Department of Oncology, University of Torino, Candiolo, Torino 10060 Italy; Candiolo Cancer Institute - FPO, IRCCS, Candiolo, Torino 10060 Italy; FIRC Institute of Molecular Oncology (IFOM), Milano, 20139 Italy

## Abstract

DNA fragments released from cancer cells into the blood can be used to generate molecular profiles of tumors. Non-invasive 'liquid biopsies' can be used to monitor minimal residual disease and detect the emergence of drug resistance.

## Introduction

Precision oncology relies on the availability of accurate molecular profiles of individual tumors, which are currently determined by genetic analysis of DNA extracted from neoplastic tissue. Sampling tumor tissue from surgical specimens or biopsies has significant limitations. Biopsies provide a single snapshot in time, are subject to selection bias resulting from tumor heterogeneity, can lead to severe clinical complications and can be difficult to obtain [1-3]. Tumor cells release DNA into the blood, and this offers the opportunity to determine the genetic landscapes of solid cancer from the circulation, an approach commonly called 'liquid biopsy'. Here, we summarize applications of liquid biopsies to interrogate cancer genomics in the blood of patients in different clinical scenarios.

## Liquid biopsies, an evolving concept

**Figure 1:**
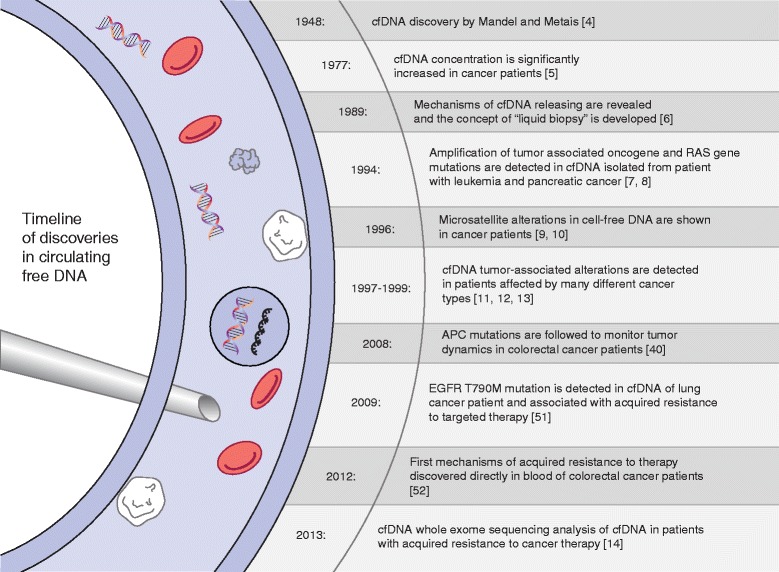
**Timeline of discoveries in cell-free DNA.** The references mentioned are [4-14,40,51,52].

In 1948, Mandel and Métais described the presence of circulating, cell-free nucleic acids (cfNAs) in human blood for the first time [4]. In 1977, further studies showed that cancer patients had higher plasma levels of circulating DNA than healthy controls [5], and Stroun and colleagues showed that circulating DNA shared some biophysical properties (such as decreased strand stability) with the DNA within cancer cells, thus suggesting that cfNA was of tumor origin [6] (Figure 1). At that time, these seminal findings attracted little attention in the scientific community. In 1994 the breakthrough finding of mutant *RAS* gene fragments in the blood of cancer patients turned the spotlight on cfDNA once again [7,8]. In 1996, microsatellite alterations in cfDNA were reported in cancer patients [9,10] and over the past two decades several types of cfNAs (such as DNA, mRNA and microRNAs) have been detected in the blood of cancer patients (Figure 1) [11-13]. In recent years, our ability to analyze cfDNA has improved rapidly, thanks to enhancements in technologies that enable detection of mutant alleles with very high specificity and sensitivity. The latest development involved applications of next-generation sequencing to tumor cfDNA, which allows mutational profiling of the entire coding sequence (exome) of cancer cells from the blood [14].

Significantly higher cfDNA concentrations are present in serum than in plasma [15-20]. The increased level of cfDNA in serum is due to the clotting of white blood cells in the collection tube, leading to their lysis [17,19,20]. As a consequence, cfDNA in serum is 'diluted' by genomic DNA released from white blood cells. Accordingly, plasma is a better source of specific tumor cfDNA [21,22].

In general, concentrations of cfNAs are higher in individuals with cancer than in healthy controls, and levels are further increased in metastasis. Both tumor-derived and normal germline cfNAs are released into the blood, and the proportion of tumor-derived cfNA is broadly related to the extent of the disease [1]. The amount of cfNA is also influenced by clearance, degradation and other physiological filtering events in the blood and lymphatic circulation [23-25]. Nucleic acids are cleared from the blood by the liver and kidneys and have a variable half-life in the circulation, ranging from 15 minutes to several hours [23]. The release of nucleic acids into the blood is not fully understood but is probably related to the fast turnover of cancer cells and the ensuing apoptosis or necrosis [24,25]. Numerous studies show that virtually all living cells actively release DNA fragments [26]. The presence of DNA and RNA in exosomes, vesicles that are actively released by multiple cell types (including neoplastic cells), is also well documented [27-29].

It has been estimated that a tumor that weighs 100 g (which corresponds to 3 × 10^10^ cancer cells), may release up to 3.3% of the DNA present in the cancer cells into the blood every day [30]. The size of tumor cfDNAs detected in the blood of cancer patients is highly variable: most are 70 to 200 base pairs long but fragments of up to 21 kilobases have also been detected [31]. In patients, the amount of cfDNA ranges between 0 and >1,000 ng/ml of blood, with an average of 180 ng/ml [32-35]. Monitoring cfDNA concentrations *per se* does not seem to be useful for diagnostic purpose because several physiological and pathological conditions besides cancer can lead to great changes in cfDNA levels. For instance, pro-inflammatory diseases, such as liver cirrhosis, hepatitis, systemic lupus erythematous or rheumatoid arthritis [36,37], all lead to the release of high levels of cfDNA.

In a recent study, levels of tumor cfDNA were evaluated in 410 patients with solid tumors [38]. The fraction of patients with detectable levels of cfDNA varied among tumor types. Most patients with stage III ovarian or liver cancer, as well as most patients with neuroblastoma or melanoma, had measurable levels of cfDNA. In addition, most patients with metastatic cancers of the pancreas, bladder, colon, stomach, breast, liver, esophagus or head and neck had measurable levels of cfDNA. Notably, less than 50% of the patients with medulloblastomas or metastatic cancers of the kidney, prostate or thyroid, and less than 10% of patients with gliomas harbored detectable cfDNA. A likely explanation for the low amount of cfDNA in patients with tumors localized to the central nervous system is the presence of the blood-brain barrier [38].

### Molecular profiles of solid cancer from cfDNA

Defining the tumor genotype of an individual patient is becoming part of the standard of care for a significant proportion of cancer patients. Genotyping can be used in several settings: to categorize the type and the stage of cancer, to establish the aggressiveness of the disease, to tailor treatment and to monitor evolution of the cancer during therapy.

Currently, the genotype of a tumor is established from tumor tissue, which is obtained through surgically or radiologically guided biopsies. However, sampling tumor tissue in this way has significant, inherent limitations. Molecular profiling of tumor tissue provides a single snapshot in time, is subject to selection bias owing to tumor heterogeneity and does not capture the complexity of the disease if multiple metastatic lesions are present [1-3,39].

When a comprehensive analysis of the overall disease is required or when tissue specimens are difficult to obtain or are unavailable, liquid biopsies are an attractive alternative option. This is because circulating tumor DNA fragments contain identical genetic defects to those in the tumors themselves and virtually all cancer-related molecular alterations can be detected in cfDNA. These include somatic point mutations, loss of heterozygosity (LOH), translocations, gene copy number changes and DNA methylation changes. For example, oncogenic alterations such as mutations in *KRAS* (Kirsten rat sarcoma viral oncogene homolog), *BRAF* (B-Raf proto-oncogene, serine/threonine kinase), *PIK3CA* (phosphatidylinositol-4,5-bisphosphate 3-kinase, catalytic subunit alpha) and *EGFR* (epidermal growth factor receptor) can be readily detected in the blood of patients with lung, colorectal, breast or pancreatic cancer, or melanoma. Mutations that inactivate common tumor suppressor genes such as *APC* (adenomatous polyposis coli) and *TP53* (tumor protein p53) have also been detected in the plasma of cancer patients [30,40]. Gene copy number variations, such as amplification of *EGFR*, *HER2* (human epidermal growth factor receptor 2) and *MET* (MET proto-oncogene, receptor tyrosine kinase), can also be detected in the blood [41-44].

**Figure 2:**
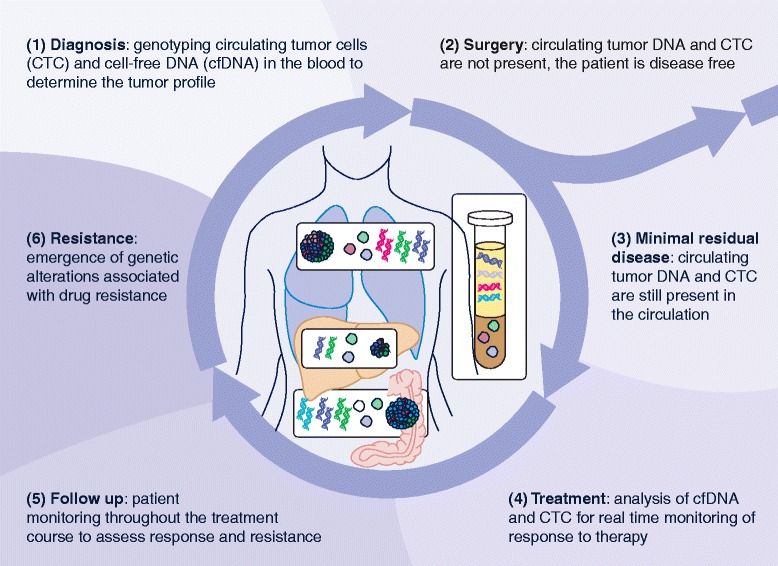
**Clinical applications of cell-free DNA analysis.** cfDNA can be used in **(1)** diagnosis **(2,3)** to detect residual disease after surgery, **(4)** to monitor the response to therapy and **(5)** follow-up, and **(6)** to detect resistance. cfDNA, cell-free DNA; CTC, circulating tumor cells.

Any tumor-specific molecular alteration can be detected in the blood of patients with cancer, and profiling of cfDNAs from plasma or serum has been proposed for several clinical applications (Figure 2), the most promising of which we discuss in the following sections.

## Clinical applications of liquid biopsies

### Monitoring minimal residual disease

Surgery with curative intent is used for many cancer patients. A central question is whether further therapy is needed after surgical removal of the cancer. Currently, predicting which patients are disease-free after surgery (cured) and those who have residual disease depends largely on clinical and pathological parameters (mainly the tumor-node-metastasis staging system). There is currently no effective diagnostic means to distinguish between those patients that are disease-free and those with minimal residual disease that cannot be detected by clinical examination or imaging. Undetected, untreated minimal residual disease gives rise to recurrences. As a result, patients with high-risk clinical and pathological criteria are indiscriminately treated with adjuvant chemotherapy. Unfortunately, this therapy is frequently not needed because the patient would have been cured with surgery and/or radiotherapy alone.

Liquid biopsies have great potential in this scenario. The detection of tumor cfDNA following surgery/radiotherapy would be an indicator of residual disease, and hence liquid biopsies could be used to identify those patients who would benefit from adjuvant therapy while sparing those who would not benefit from unnecessary treatment. Detection of tumor cfDNA after completion of surgery/radiotherapy would indicate the presence of micro-metastasis and much greater risk of relapse, indicating a need for adjuvant therapy. Moreover, the detection of tumor cfDNA would indicate early relapse and could potentially be used to tailor targeted therapy to the patient [45] (Figure 2).

Recently, Beaver and colleagues [46] studied tumor cfDNA to detect *PIK3CA* mutations and to monitor residual disease after surgery in patients with early stage breast cancer. Primary breast tumors and matched pre- and post-surgery blood samples were collected from early-stage breast cancer patients. The study was conducted prospectively, which adds considerable relevance to the findings. Droplet digital PCR (ddPCR) was used to assess the presence of *PIK3CA* mutations (in exon 9 and 20) in the blood of estrogen-receptor- and progesterone-receptor-positive early-stage breast cancer patients. DNA extracted from 30 tumor samples was analyzed by Sanger sequencing for common *PIK3CA* mutations; 10 samples were found to carry *PIK3CA* mutations. The same 30 samples were analyzed by ddPCR, which identified five more patients carrying *PIK3CA* mutations. Plasma cfDNA was then isolated from pre- and post-operative blood samples. In 14 of the 15 samples with mutations, those mutations were detected in pre-surgery plasma samples by ddPCR, whereas no mutations were found in the pre-surgery samples in which post-surgery samples showed no mutations. The sensitivity and specificity of cfDNA analysis to detect mutations were thus remarkable (93.3% and 100%, respectively). Among 10 of the patients with mutation-positive pre-surgery tumor cfDNA, analysis of post-surgery plasma detected *PIK3CA* mutations in five. These patients had no other clinical evidence of disease by any radiological examination the patients were subjected to. Overall, the work of Beaver and colleagues [46] presents the first evidence that a 'liquid biopsy' approach can be used to identify patients with breast cancers at early stages and at risk of recurrence after surgery.

Liquid biopsy can also be used to monitor tumor dynamics in a cancer patient. For example, assessing the fluctuations of an *APC* mutation, which is known to be the initiating lesion in colorectal cancer tumorigenesis, during the follow-up of a patient provides a measure of the systemic tumor burden, because levels of the mutation in circulating DNA decrease following surgery and increase as new lesions appear (as detected by radiological examination) [40].

### Tracking emergence of drug resistance

In the past decade, drugs that target genetically defined tumor vulnerabilities (such as *EGFR* or *BRAF* mutations) have shown remarkable effectiveness [47]. Acquired resistance, which is observed in virtually all patients following treatment with anticancer drugs, limits the utility of these drugs and is a substantial challenge to the clinical management of cancer patients. Accordingly, understanding the molecular basis and development of acquired resistance is key to trying to overcome it. Analysis of tumor cfDNA provides a valuable opportunity to repeatedly monitor molecular response during targeted treatment and detect early evidence of emerging resistance (such as specific mutations) and tumor recurrence, as described below [14,40,48-50].

Tracking the genomic evolution of cancer during therapy is an unmet clinical need. As discussed above, tissue biopsies reveal only a fraction of the overall disease heterogeneity, especially in patients with metastatic disease. It is fair to say that no effective means currently exists to assess the molecular evolution of the overall disease during the course of therapy in patients with multiple metastatic lesions in distinct organs.

In contrast, tumor cfDNA analysis enables early identification of molecular changes associated with drug resistance and can be easily repeated multiple times in one patient.

**Figure 3:**
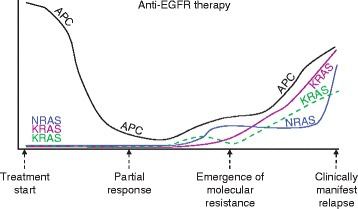
**Schematic representation of clonal evolution in patients treated with anti-EGFR monoclonal antibodies.** The lines are examples of the likely levels of mutated cfDNA detected at different stages of treatment. *APC* mutation (black line) reflects tumor burden, which decreases after treatment but increases again when resistance develops. Different *KRAS* and *NRAS* mutations (green, purple and blue lines) are detected in cfDNA at the acquisition of resistance, before relapse is clinically manifest.

Analysis of tumor cfDNA in plasma samples obtained before and after treatment can be used to provide a global picture of the molecular genetics of a patient's tumor. This molecular picture includes dynamic changes in the mutation profile that occur during therapy, as well as the heterogeneity that emerges as a result of therapeutic selective pressure. Understanding the mechanisms of acquired resistance to targeted agents at the molecular level can be used to plan new therapeutic approaches with drugs that will suppress the expansion of the clones that are responsible for most of the current failures of medical treatments. This knowledge could provide the early adoption of alternative therapies before resistance is detected and clinically manifest (Figure 3).

## Conclusions and future directions

Liquid biopsies can improve the effectiveness of precision oncology, with potential benefits to patients and healthcare systems. Research on liquid biopsies has seen enormous development in the past 5 years; this has led to a proliferation of publications, most of which involve very small numbers of patients. None of these studies presented definitive evidence as to how the analysis of tumor cfDNA may be clinically relevant or applicable [14,38,41-44,46,48-52]. What the field needs are well-controlled clinical studies, involving extensive cohorts of patients, in which liquid biopsies are used to address clinically relevant questions. We propose that these studies should focus on two questions: the development of non-invasive tests to detect minimal residual disease in patients affected by solid cancers; and the use of liquid biopsies to monitor the emergence of molecular resistance to anticancer therapies before relapse is clinically manifest. This could lead to the early adoption of further lines of therapy.

## Abbreviations

APC: Adenomatous polyposis coli
BRAF: B-Raf proto-oncogene, serine/threonine kinase
cfDNA: cell-free DNA
cfNA: cell-free nucleic acid
ddPRC: droplet digital PCR
HER2: Human epidermal growth factor receptor 2
KRAS: Kirsten rat sarcoma viral oncogene homolog
LOH: loss of heterozygosity
MET: MET proto-oncogene, receptor tyrosine kinase
PIK3CA: Phosphatidylinositol-4,5-bisphosphate 3-kinase, catalytic subunit alpha
TP53: Tumor protein p53
